# Analysis of pressure-activated Piezo1 open and subconductance states at a single channel level

**DOI:** 10.1016/j.jbc.2024.107156

**Published:** 2024-03-11

**Authors:** Ghanim Ullah, Elena D. Nosyreva, David Thompson, Victoria A. Cuello, Luis G. Cuello, Ruhma Syeda

**Affiliations:** 1 Department of Physics, University of South Florida, Tampa, Florida, USA; 2 Department of Neuroscience, University of Texas Southwestern Medical Center, Dallas, Texas, USA; 3 Department of Cell Physiology and Molecular Biophysics, TTUHSC, Lubbock, Texas, USA

**Keywords:** Piezo1, mechanically activated ion channels, patch clamp recordings, subconductance level, single channel model

## Abstract

Mechanically activated Piezo1 channels undergo transitions from closed to open-state in response to pressure and other mechanical stimuli. However, the molecular details of these mechanosensitive gating transitions are unknown. Here, we used cell-attached pressure-clamp recordings to acquire single channel data at steady-state conditions (where inactivation has settled down), at various pressures and voltages. Importantly, we identify and analyze subconductance states of the channel which were not reported before. Pressure-dependent activation of Piezo1 increases the occupancy of open and subconductance state at the expense of decreased occupancy of shut-states. No significant change in the mean open time of subconductance states was observed with increasing negative pipette pressure or with varying voltages (ranging from −40 to −100 mV). Using Markov-chain modeling, we identified a minimal four-states kinetic scheme, which recapitulates essential characteristics of the single channel data, including that of the subconductance level. This study advances our understanding of Piezo1-gating mechanism in response to discrete stimuli (such as pressure and voltage) and paves the path to develop cellular and tissue level models to predict Piezo1 function in various cell types.

Piezo1 is a mechanically activated, cation selective channel that opens in response to physical stimuli such as pressure, tension, and sheer stress. The importance of Piezo1 function is underscored by various mouse models and functional data that established its role in vascular development (1, 2), bone homeostasis (3), blood cell volume regulation (4, 5), and related disorders such as xerocytosis (6) among others (7, 8, 9, 10). Additionally, the biophysical properties of Piezo1 channels are well studied using cryo-EM and patch-clamp techniques. Structure-function studies of Piezo1 show a central ion conduction pore, with three peripheral blades giving a channel a bowl-like conformation in a closed-state (11, 12, 13), a flattened conformation in a presumed open-state in a stretched membrane (14, 15), distinct unitary conductance (2, 16, 17), and fast inactivation kinetics (18, 19, 20). Despite this valuable information, not much is known about pressure-dependent gating transitions (caused by direct stretch of the membrane), the kinetic scheme that Piezo1 follows at the single channel level, and whether channel dwells into any sub-state while transitioning from closed to open-state.

Many ion channels exhibit subconductance levels (SCLs), such as voltage gated potassium channels (21), NMDA receptors (22), glutamate receptors (23), and stretch-activated OSCA channels (24). The functional analysis of ion channels is usually focused on properties, such as current-amplitude, unitary or slope conductance, open and closed dwell times, single channel open probability, and inactivation kinetics. Investigating SCLs of ion channels can be challenging mainly because of two reasons: (i) the inherently low occupancy and occurrence of SCLs and poor signal-to-noise ratio, leading to broad peak current-amplitude histograms and (ii) SCLs can be extremely short lived (<1 ms), which could be interpreted as an artifact produced by very fast switching observed under limited bandwidth of the patch clamp amplifier (25).

In light of the literature where many ion channels do not follow a simple two state model (open and closed state) but also exhibit more than one open state conductance (26), we define here the criteria for recognizing subconductance state as follows: i) sub-states have lower conductance than the fully open state, ii) sub-states must be distinguishable from second channel population if more than one channel is present in a patch, (iii) less frequently occurring conductance levels than a fully open state, iv) observation of transitions from one conductance level to the other. With these parameters in mind, here we sought out to perform comprehensive single channel analysis to identify SCLs of pressure-activated Piezo1 channels and how are they modulated by external stimuli such as pressure and voltages. We compared occupancy, dwell times, and current-amplitude of Piezo1 shut-state (sh) *versus* sub-state (s) and open-state (o). Our findings suggest that steady-state open probability and kinetic schemes of Piezo1 are most dependent on applied pressure. The steady-state open probability and occupancy of subconductance state is increased in response to increasing negative pressure in the patch-pipette. We propose that Piezo1 gating at increasing negative pressures utilizes subconductance state and may aid in regulating ionic influx in a cell under steady-state conditions.

Our observation of the SCLs also warrants the development of a new single channel model for Piezo1 gating, as the previous models only focused on shut, open, and inactivated states (27, 28). Furthermore, these models did not incorporate the dependence of the channel’s kinetics on voltage. The transition rates were derived at discrete pressure values, which precludes using these models to computationally investigate the role of Piezo1 in the cell’s function where pressure and voltage changes dynamically. Thus, we used the theory of aggregated binary reversible Markov chains to derive a single-channel model for Piezo1 *via* an iterative, data-driven approach (29, 30). We find this approach simpler and more intuitive because it separates thermodynamic quantities (equilibrium occupancies or constants) from kinetic quantities (reaction fluxes or reaction rates). Our model closely fits experimental observations and allows for future studies on determining the significance of Piezo1 SCLs in the cell’s function.

## Results

**Figure 1:**
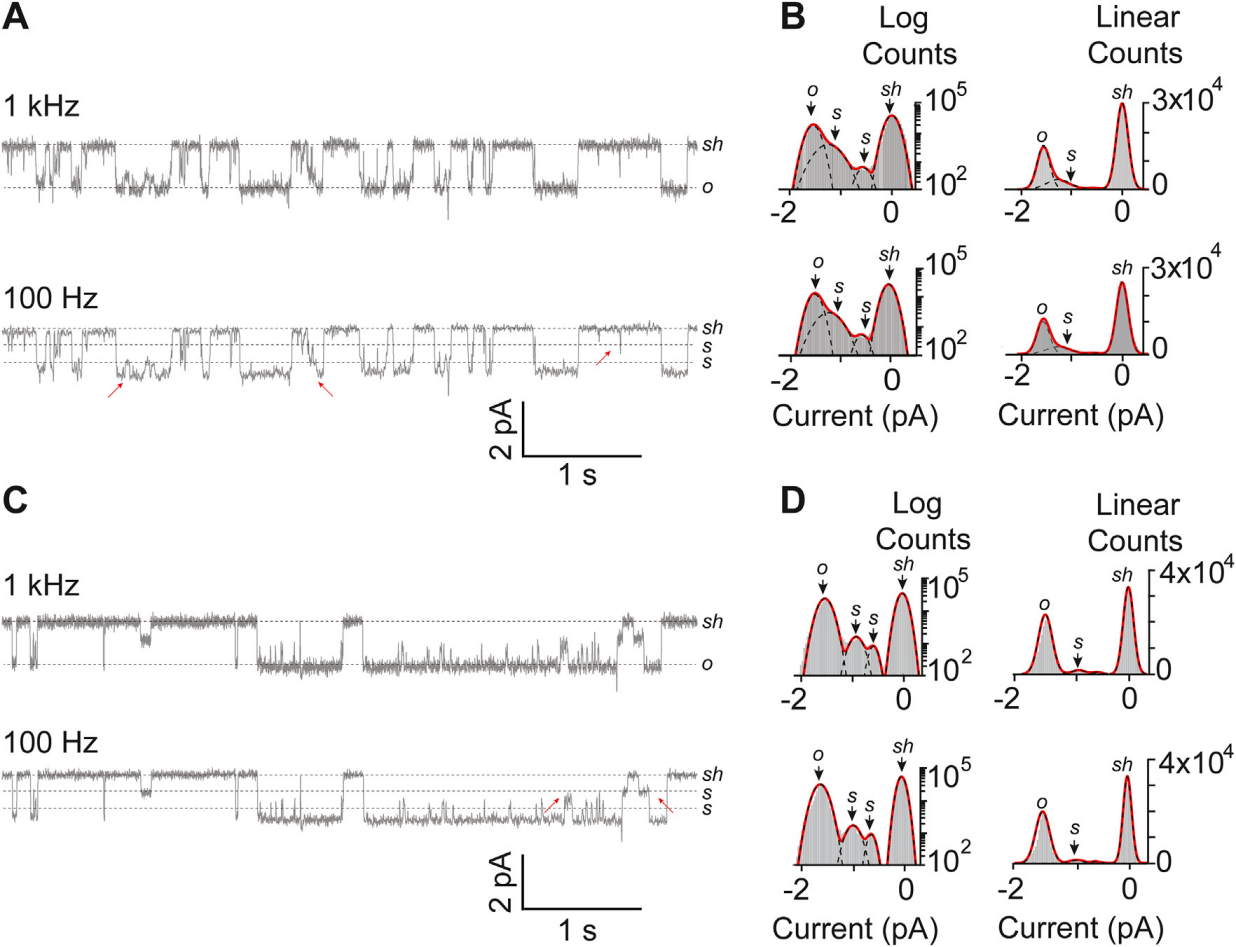
**Identification of subconductance state in Piezo1 channels recorded from transfected HEK293T**^**ΔP1**^**cells and in asymmetric lipid bilayers.***A*, heterologously expressed Piezo1 single-channel activity recorded from HEK293T^ΔP1^ cells in a cell-attached mode at −60 mV and −30 mm Hg applied pressure. *B*, respective, all-point current-amplitude histograms with y-axis presented as logarithmic scale and linear scale. The pipette solution contained (mM) the following: 130 Na^+^, 1 Mg^++^, and 1 Ca^++^; the bath solution contained (mM) the following: 140 K^+^ and 1 Mg^++^. *C*, single-channel activity of purified Piezo1 reconstituted in asymmetric droplet lipid bilayers. *D*, respective, all-point current-amplitude histograms with y-axis presented as logarithmic scale and linear scale. The droplet solution contained (in mM) the following: 130 KCl, 10 Hepes pH 7.4. *sh*, *s*, and *o* denote the shut-state, sub-state, and open-state of the channel. Representative traces (*A* and *C*) were filtered at 1Khz and 100 Hz frequency for display and comparison of all-point histograms.

In this study, we perform cell-attached recordings of heterologously expressed mouse Piezo1 in HEK 293T Piezo1 KO cells. Application of direct negative pressure to the membrane patch evoked single and multiple channels (ranging from 1 to 5 channels in a patch). We use smaller diameter pipette with 4 to 6 MΩ resistance and 0.5 to 1 μg of DNA for transient transfection to avoid macroscopic currents. This strategy usually yielded one to two channels in a patch. While inactivation is the hallmark of Piezo1 in response to mechanical stimulus (which we observe in the patches containing >5 channels), here the focus is on the steady-state currents. Careful examination of the raw data and analysis *via* different idealization methods revealed prominent SCLs in addition to a distinct shut- and open-state (Fig. 1*A*, upper panel).

The raw data acquired at −60 mV and at negative pipette pressure (−30 mm Hg for ∼30 s) was used to construct all-point current-amplitude histograms. To assess whether SCLs are statistically prominent enough to appear as a distinct population, the histograms were fitted with the Gaussian function with two, three, or four parameters using least square fitting method. The best fit was achieved with four variables (Fig. 1*B*) when the data points were constructed as a logarithmic scale ranging from 1 to 100,000 points or as a linear scale. The four parameters in the Gaussian function are attributed to the shut-state (0 pA), fully open-state (mean ± SD = −1.5 ± 0.05 pA), and two in-between SCLs (mean ± SD = −0.5 ± 0.03 pA and −1.1 ± 0.02 pA respectively).

To determine whether sublevels are real gating transitions and not an artifact of incomplete or fast transitions, the data was filtered to extreme frequencies (100 Hz). As expected, this filtering frequency only decreased the baseline noise, but the SCLs occurrences were intact and visually detected in a 30 s steady-state recording (Fig. 1*A*, lower panel). The histograms constructed from the 100 Hz–filtered data mirror the histograms acquired from 1 kHz frequency data, suggesting that SCLs are true property of heterologously expressed Piezo1 channels and not the noise that could be filtered at extreme frequencies. Additionally, we asked whether the occurrence of SCLs is dependent on cellular factors or is it an inherent property of Piezo1 channels? To accomplish this, we purified Piezo1 from HEK 293T cells and reconstituted in asymmetric droplet lipid bilayers to obtain single-channel currents. This strategy has previously been valuable to show that Piezo1 is a *bona fide* pore-forming ion channel capable of conducting ion without any cellular components (16, 17). All-point current-amplitude histograms of the purified proteins clearly showed occurrence of sublevels (Fig. 1, *C* and *D*), in line with the data acquired from cells, validating that Piezo1 has a kinetically stable and recognizable SCLs despite the stimuli used to evoke channel openings (applied pressure in cells and membrane tension in asymmetric lipid bilayers). To summarize, subconductance state is the intrinsic property of Piezo1 channels that has not been explored before.

### Effect of pressure on Piezo1 gating transitions

**Figure 2:**
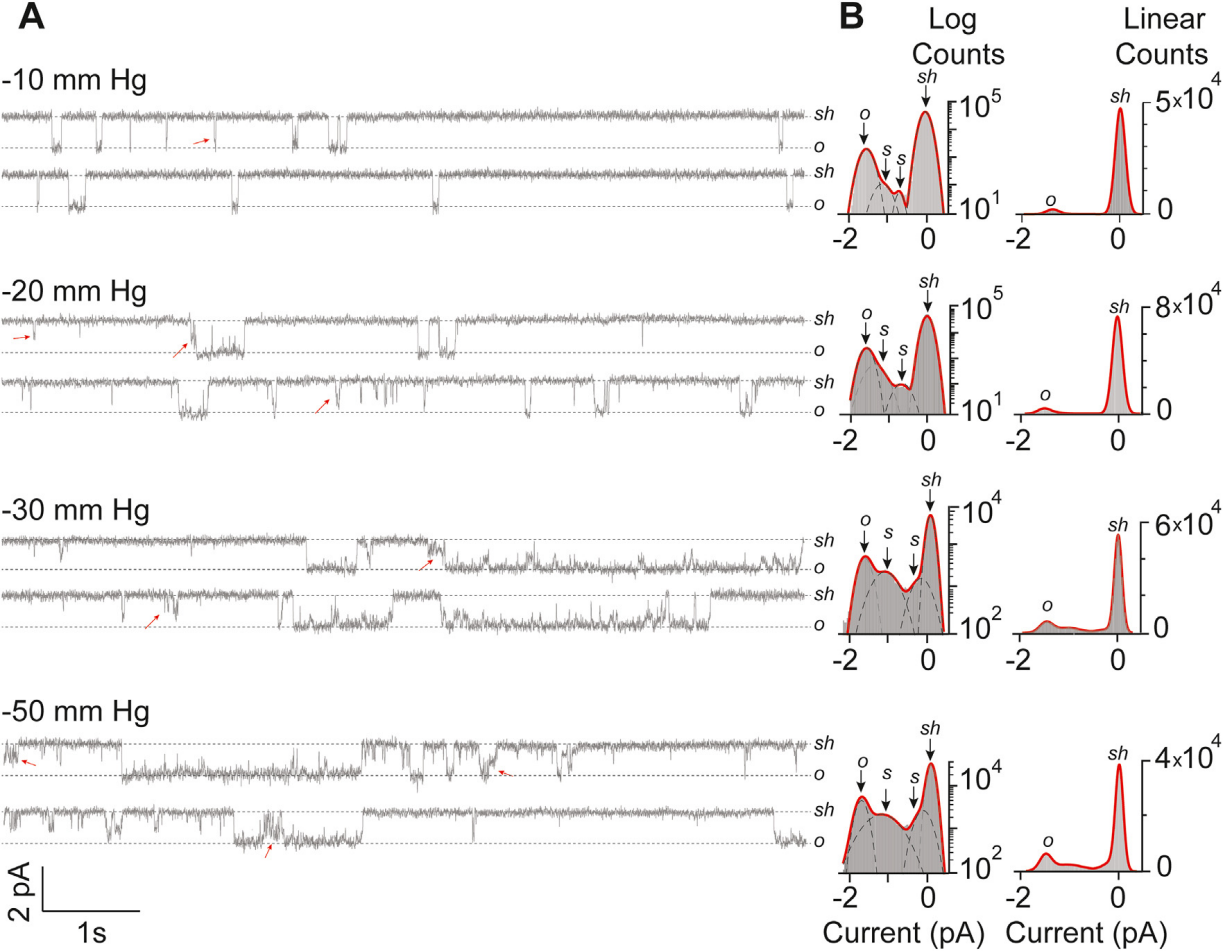
**Effect of negative pressure on Piezo1 Sub-levels.***A*, heterologously expressed Piezo1 single-channel activity (∼16 s) recorded from HEK293T^ΔP1^ cells in a cell-attached mode at a constant voltage of −60 mV and at indicated applied negative pressures. *Sh* and *o* represent the shut state and open state of the channel; *red arrows* point out examples of sub states in between open and shut state. *B*, respective all-point current-amplitude histograms with y-axis presented as logarithmic scale and linear scale. *sh*, *s*, and *o* denote the shut-state, sub-state, and open-state of the channel.

**Figure 3:**
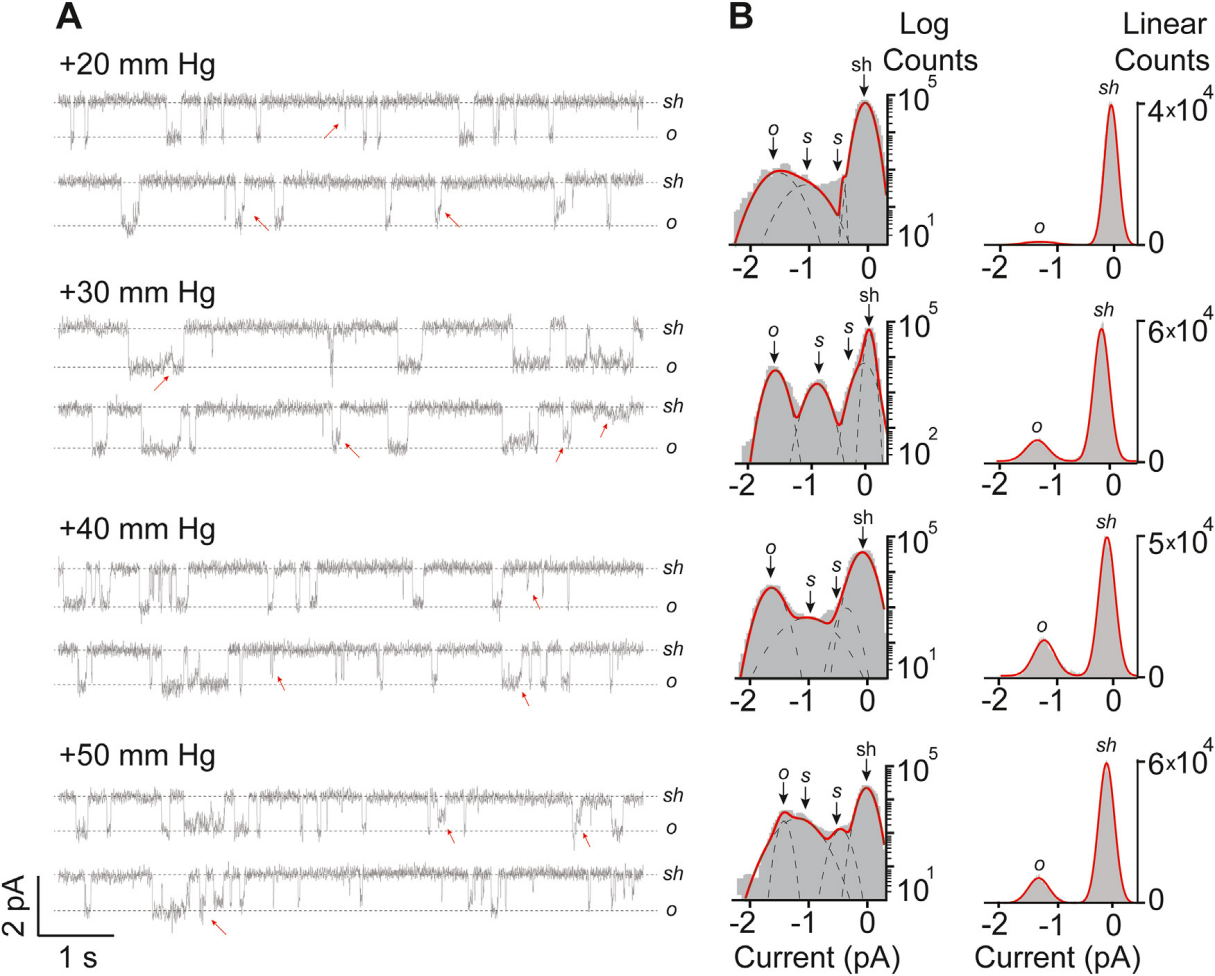
**Effect of positive pressure on Piezo1 Sub-levels.***A*, heterologously expressed Piezo1 single-channel activity (∼15 s) recorded from HEK293T^ΔP1^ cells in a cell-attached mode at a constant voltage of −60 mV and at indicated positive applied pressures. *Sh* and *o* represent the shut state and open state of the channel; *red* arrows point out examples of sub states in between open and shut state. *B*, respective all-point current-amplitude histograms with y-axis presented as logarithmic scale and linear scale. *sh*, *s*, and *o* denote the shut-state, sub-state, and open-state of the channel.

Once we established the occurrence of SCLs in Piezo1 both from the purified proteins and heterologous expression in cells, next we focused on the role of applied pressure on gating transitions from various states (such as shut to open or shut to SCLs). The data was collected under sustained pressure pulses of −10, −20, −30, −50, and −60 mm Hg for 30 s each. Steady-state single-channels were analyzed similarly as established for Figure 1. Clearly, not all transitions from shut-state reached directly to the fully open-state, hence populating the occupancy of SCLs (Fig. 2*A*). The current-amplitude histograms were fitted by Gaussian function indicating the shut, open, and in between SCLs, revealing that the frequency and the occurrence of SCLs increases with increasing negative pressure (Fig. 2*B*). Furthermore, we asked whether negative applied pressure is the only trigger for subconductance levels in cells? Therefore, we applied positive pressures (ranging from +20 mm Hg to +50 mm Hg) to cell-attached patches and obtained Piezo1 single channel data (Fig. 3*A*). All-point current-amplitude histograms showed occurrence of subconductance states when fitted with Gaussian functions, where shut-state is observed at 0 pA, fully open-state at, mean ± SD = −1.4 ± 0.06 pA, and two in-between SCLs were observed at mean ± SD = −0.4 ± 0.03 pA and −0.9 ± 0.04 pA, respectively (Fig. 3*B*). These experiments established that subconductance states are the inherent property of Piezo1 ion channels regardless of the direction of applied pressure and the geometry of the patch membrane (31).

**Figure 4:**
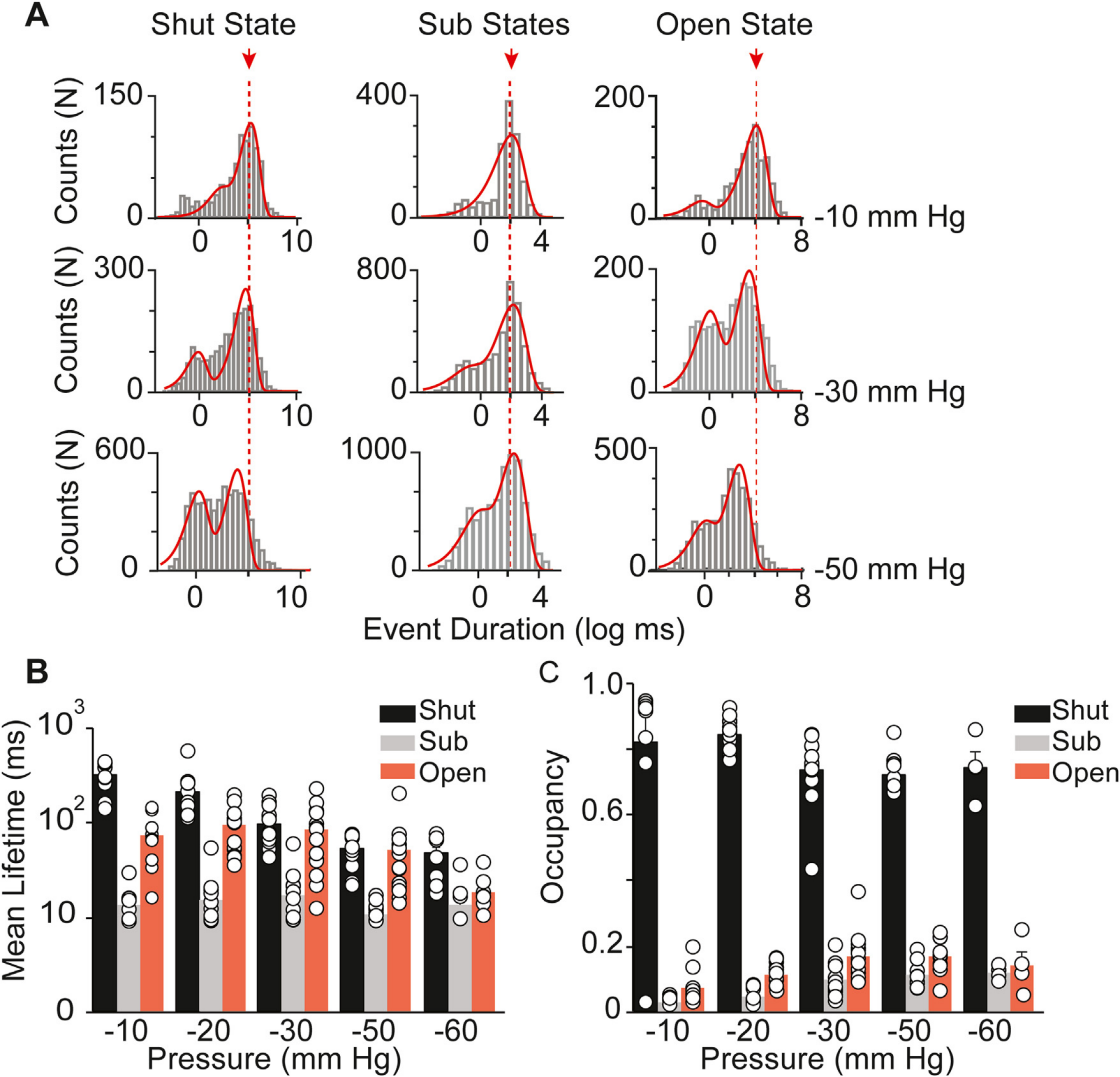
**Dwell time distribution of Piezo1 in different states as a function of pressures.***A*, dwell time histograms of Piezo1 in a shut-state, sub-state, and open-state at various pressures; −10 mmHg (*upper row*), −30 mmHg (*middle row*), and −50 mm Hg (*bottom row*). *B*, mean lifetimes of Piezo1, either in shut-state (*black*), sub-state (*gray*), and open-state (*red*) at indicated pressure values. One way ANOVA *p* = 0.0029 for shut state, *p* = 0.6083 for sub state, and *p* = 0.0668 for open state. *C*, occupancy of Piezo1 either in shut-state (*black*), sub-state (*gray*), and open-state (*red*) at indicated pressure values. One way ANOVA *p* = 3.8e-7 for shut state, *p* = 8.0e-7 for sub state, and *p* = 0.0004 for open state. Significant difference when *p* < 0.05 or no significance difference when *p* > 0.05. Data was acquired at constant voltage −60 mV. Scattered bar graphs represent data as mean ± SD acquired from individual cell-attached patches (n = 11 at −10 mm Hg, n = 14 at −20 mm Hg, n = 14 at −30 mm Hg, n = 14 at −50 mm Hg, and n = 8 at −60 mm Hg).

Next, we analyzed mean lifetimes of Piezo1 channels in each state by constructing dwell time histogram and fitting the Gaussian function to the data (Fig. 4*A*). The SCLs are indeed fast and short-lived transitions, whereas channel spent significant time in the shut-state and open-state when assayed at −60 mV and at various applied pressures (Fig. 4, *A* and *B*). As noted previously (27), we observed clear effect of pressure on shut dwell times distributions (mean shut time decreases with increasing pressure), but the SCLs and open-state dwell times remains the same in response to increasing negative pressure (no statistical significance *p* > 0.05, Table 1). To assess the frequency and occurrence of SCLs in response to pressure, we calculated occupancy of the channel in each state; shut, SCLs, and open (Fig. 4*C*). The calculated occupancy of Piezo1 at −60 mV and at −30 mm Hg pressure for both fully open-state and SCLs were mean ± SD = 0.17 ± 0.02 and 0.09 ± 0.01, respectively. The occupancy of full openings and SCLs are in good agreement with the calculated over all open probability of the channels. This analysis suggests that the increase in pressure does not have a significant effect on SCLs dwell times and open-state dwell times but promote frequent shut to SCLs transitions, pressure-dependent openings, hence decreasing the shut-state dwell times (Table 1).

**Table 1:** Piezo1 occupancy and mean lifetimes as functions of negative pressure and voltage

Occupancies *versus* pressure (from −10 mm Hg to −60 mm Hg)			
State	F value	*p* value	Effect
Shut	12.75	3.82364e-7	Decrease
Sub	11.97	8.00828e-7	Increase
Open	6.22	0.0004	Increase


Mean lifetimes *versus* pressure (From −10 mm Hg to −60 mm Hg)			
State	F value	*p* value	Effect
Shut	4.67	0.0029	Decrease
Sub	0.68	0.6083	No effect
Open	2.36	0.0668	No effect


Occupancies *versus* voltage (From −40 mV to −100 mV)			
State	F value	*p* value	Effect
Shut	7.93	0.0006	Increases
Sub	5.62	0.004	Decreases
Open	7.73	0.0007	Decreases


Mean lifetimes *versus* voltage (From −40 mV to −100 mV)			
State	F value	*p* value	Effect
Shut	0.84	0.4846	No effect
Sub	1.61	0.2099	No effect
Open	1.49	0.2384	No effect

One-way ANOVA analysis showing the significance difference (*p* < 0.05) or no significance (*p* > 0.05) of Piezo1 states (shut, sub, and open) at various applied pressures (from −10 to −60 mm Hg) and voltages (from −40 to −100 mV).

**Figure 5:**
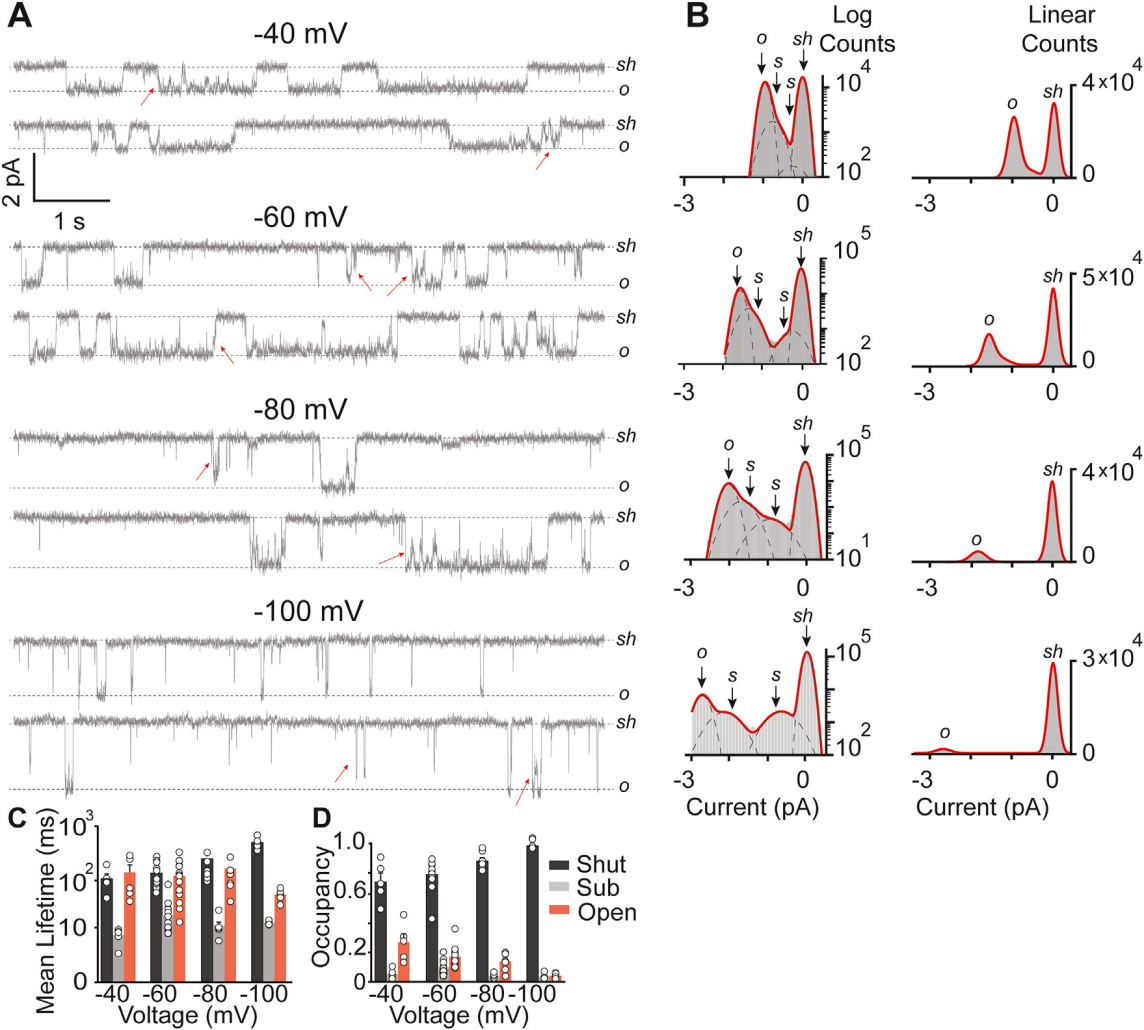
**Effect of voltage on Piezo1 closed, sub-state, and open-state.***A*, heterologously expressed Piezo1 single-channel activity (∼16 s) recorded from HEK293T^ΔP1^ cells in a cell-attached mode at a constant pressure of −30 mm Hg and at indicated applied voltages. *Sh* and *o* represent the shut state and open state of the channel; *red* arrows point out examples of sub states in between fully open and shut state. *B*, respective, all-point current-amplitude histograms with y-axis presented as logarithmic scale or linear scale. *sh*, *s*, and *o* denote the shut-state, sub-state, and open-state of the channel. *C*, mean lifetimes of Piezo1 either in a shut-state (*black*), sub-state (*gray*), and open-state (*red*) at indicated applied voltages. One way ANOVA *p* = 0.4846 for shut state, *p* = 0.2099 for sub state, and *p* = 0.2384 for open state. *D*, occupancy of Piezo1 either in a shut-state (*black*), sub-state (*gray*), and open-state (*red*) at indicated applied voltages. One way ANOVA *p* = 0.0006 for shut state, *p* = 0.004 for sub state, and *p* = 0.007 for open state. Significant difference when *p* < 0.05 or no significance difference when *p* > 0.05. The data was acquired at constant pressure of −30 mmHg. Scattered bar graphs represent data as mean ± SD acquired from individual cell-attached patches (n = 6 at −40 mV, n = 14 at −60 mV, n = 7 at −80 mV, and n = 6 at −100 mV).

### Effect of voltage on Piezo1 gating transitions

**Figure 6:**
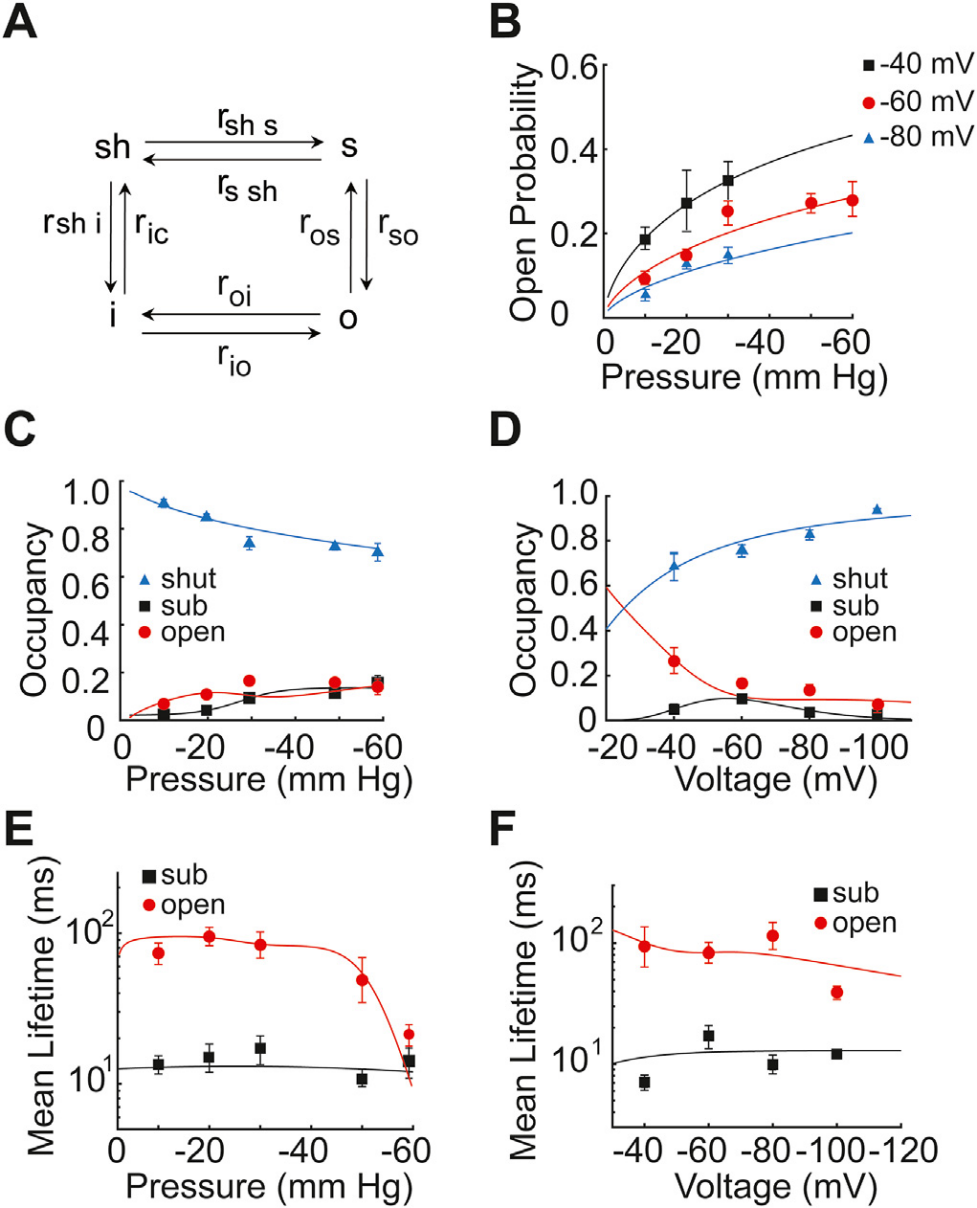
**A four-state model for the gating kinetics of Piezo1 as functions of pressure and voltage.***A*, model scheme used where sh, s, o, and i represent a shut-state, sub-state, main open-state, and inhibited-state, respectively, and r_xy_ represents the transition rate between state x and y. *B*, fits to the observed P_o_ as a function of pressure at a membrane potential of −40 mV (*black*), −60 mV (*red*), and −80 mV (*blue*). The probabilities of s, o, sh, and i combined as functions of (*C*) pressure and (*D*) voltage. Mean lifetimes of sub (*black*) and open (*red*) as functions of (*E*) pressure and (*F*) voltage. In (*B*–*F*), symbols represent experimentally calculated values (from Figs. 2, 4, and 5) and lines represent model fits.

In addition to pressure, we investigated the effect of voltage on pressure-dependent transition. Sustained −30 mm Hg of pressure was applied to the patch of cell membrane while changing the potential from −40, −60, −80 to −100 mV (∼30 s of channel activity was recorded at each potential). Single-channel raw data and the subsequent all point current-amplitude histograms again showed the presence of SCLs in addition to shut and fully open-state (Fig. 5, *A* and *B*) (28, 32). At various applied voltages, the dwell times of shut, open, and SCLs are not affected, suggesting no direct correlation in response to voltage (Fig. 5*C* and Table 1). Surprisingly, the occupancies of shut, open, and SCLs are all dependent on applied voltages, reminiscent of voltage-dependent inactivation of Piezo1 channels in a whole cell configuration. In line with the reduced over-all open probability (from −40 mV to −100 mV), the occupancy of sub- and open-states also changes with membrane potentials (Fig. 5*D* and Table 1). Under near resting membrane potential of −60 mV, the probability of Piezo1 opening is P_o_ = 0.25 ± 0.08, which then significantly increases at −40 mV P_o_ = 0.35 ± 0.07 and decreases at −80 mV P_o_ = 0.1 ± 0.03, whereas no significant dependence was found on mean lifetimes of shut-state, sub-state, or open-state of Piezo1 in response to various voltages (Fig. 6 and Table 1), indicating that the decrease in the open probability with increasing negative voltages is due to the increase and decrease in the occupancy of shut-states and open-state, respectively.

### Kinetic model for Piezo 1 function

Our experimental observations allow us to model the gating kinetics of Piezo1 with the model structure and parameters directly inferred from the data (Fig. 6*A*). We accomplish this by working under the following major assumptions: (a) the model is a finite-state Markov chain, (b) the chain obeys detailed balance, and (c) the model should be as simple as possible. Following our approach used for inositol 1,4,5-trisphohate receptors (33, 34), instead of fitting rates directly to time traces, we separate parameters governing the “state occupancies” and “mean lifetimes” as suggested by Yang et.al, previously (35). We find this approach simpler and more intuitive because it separates thermodynamic quantities (equilibrium occupancies) from kinetic quantities (reaction rates).

The all-point current-amplitude histograms (constructed from data acquired at −60 mV and −30 mm Hg) indicate four main states: (i) shut (current = 0 pA), (ii) first SCL (current = −0.5 ± 0.03 pA), (iii) a second SCL (current = −1.1 ± 0.02 pA), and (iv) fully open-state (current −1.5 ± 0.05 pA). There is also strong evidence for the existence of an inactivated state with current = 0 pA (27, 36). The existence of two states with 0 pA current is also clear from the dwell time histogram of the shut-state (Fig. 4*A*) where two peaks are observed with distinct dwell time durations. Therefore, in this study, we exclusively used the term shut-state (sh) instead of closed-state (c). Furthermore, in our analysis, the open probability (P_o_) of a channel is defined as the fraction of time for which the channel is either gating in the SCLs or main open-state. Guided by these considerations, the P_O_ of Piezo1 channel as a function of pressure and voltage can be written as

(1) $$P_{o}\left(p,v\right)=\frac{Z_{O}}{Z_{O}+Z_{C}+Z_{I}}$$

For clarity, we use the unitless forms of pressure and voltage applied to the membrane. That is, p = -pressure (in mm Hg)/(1 mm Hg) and v = −voltage/(1 mV). Z_O_, Z_C_, and Z_I_, respectively are the unnormalized occupancies of all states with non-zero current (*i.e.*, main open-state and SCLs), closed-state, and inactivated-state with respect to the closed-state. We take the closed-state to be our reference state (*i.e.*, Z_C_ = 1) and model Z_O_ and Z_I_ as

(2) $$Z_{O}=K_{O}p^{n_{o}}v^{n_{vo}}$$

(3) $$Z_{I}=K_{I}p^{n_{i}}v^{n_{vi}}$$

The K_O_, K_I_, n_o_, n_vo_, n_i_, and n_vi_ are unknown parameters. Their values are obtained by fitting equation (1) to the observed P_O_ values at various pressure and voltage values (Fig. 6*B*) and are listed in Table 2. We remark that while our choice of functional form for Z_O_ and Z_I_ is guided by the desire to keep the model as simple as possible, one can choose other functions as well.

**Table 2:** Parameter values for the open probability (P_o_) of Piezo1 channel and occupancies of different states

Parameter	Value
K_o_	13.2231 × 10^−5^
K_i_	0.72775 × 10^−5^
n_o_	3.32759
n_i_	2.66169
n_vo_	1.93981
n_vi_	3.53799
a_1_	0.01741
b_1_	0.06011
c_1_	24.78969
d_1_	3.85956
a_2_	0.28653
b_2_	4.01913
c_2_	1.01877

As mentioned above, Z_O_ is the total unnormalized occupancy of all states with non-zero current. Similarly, $P_{O}\left(p,v\right)$ is their total normalized occupancy (or probability). Furthermore, motivated by the relatively low occupancy of the SCLs with current = −0.5 ± 0.03 pA (note the significantly smaller peak in Fig. 1*B*), we aggregated the two SCLs into one. Thus, the next step is to split $P_{O}\left(p,v\right)$ (or Z_O_) into the occupancy of the main open and aggregated SCLs (s). This is achieved by fitting the following function to the normalized occupancy of the main open-state (OC_o_) as a function of pressure and voltage (Fig. 6, *C* and *D*).

(4) $$OC_{o}=\left(a_{1}+\frac{b_{1}}{1+\exp^{\left(-\frac{p-c_{1}}{d_{1}}\right)}}\right)\;\left(\frac{1}{a_{2}\sqrt{2\pi v}}\exp^{\left(-\frac{{\left(\log\left(v\right)-b_{2}\right)}^{2}}{2{a_{2}}^{2}}\right)}v^{c_{2}}\right)$$

where a_1_, b_1_, c_1_, d_1_, a_2_, b_2_, and c_2_ come from the fit and are listed in Table 2. The normalized occupancy of the SCL (OC_S_) comes from the conservation of probability, that is,

(5) $${OC}_{s}=\max\;\left(0,P_{o}\;\left(p,v\right)\;-{OC}_{o}\right)$$

The maximum condition is used to avoid negative values of the probability. The model fits the normalized occupancies of the main open-state and SCLs as functions of pressure and voltage (Fig. 6, *C* and *D*), respectively. Since it is not possible to separate the occupancies of closed and inactivated states in the experimental data (the current in both states is 0 pA), we compare the combined normalized occupancy of both states from our model and experiment (Fig. 6*D*).

The considerations about the P_O_ and occupancies result in the four-state model shown in Figure 6*A*, where “c,” “s,” “o,” and “i" represent the shut, SCLs, open, and inactivated-state, respectively. Next, we used the mean lifetimes of the fully open-state and SCLs to determine the transition rates between different states. In our formalism, the unnormalized occupancy of a state “y” with respect to the reference state “x” is the ratio of the product of forward rates from “x” to “y” to the product of backward rates from “y” to “x,” irrespective of the path taken from “x” to “y’ (35, 37). Thus,

(6) $$Z_{s}=\frac{r_{cs}}{r_{sc}}$$

(7) $$Z_{mo\;}=\frac{r_{cs\;}\times r_{so}\;}{r_{sc\;}\times r_{os}}=Z_{S\;}\times \frac{r_{so}}{r_{os}}$$

(8a) $$Z_{I}=\frac{r_{cs\;}\times r_{so}\times r_{oi}\;}{r_{sc\;}\times r_{os}\times r_{io}\;}=Z_{mo\;}\times \frac{r_{oi}}{r_{io}}$$

Where r_xy_ is the transition rate from state “x” to state “y.” *Z*_*S*_, *Z*_*mo*_, and *Z*_*I*_ is the unnormalized occupancies of the SCL, main open-state, and inactivated-state, respectively. Note that $Z_{I}$ can also be written as

(8b) $$Z_{I}=\frac{r_{ci}}{r_{ic}}$$

However, this will lead to the same result as the model has to obey detailed balance.

The probability flux out of state is equal to the product of the unnormalized occupancy of the state with the sum of all rates going out of the state. Thus, the probability flux out of state “s” and "o" is given as

(9) $$f_{s}=Z_{s}\times \left(r_{sc}+r_{so}\right)$$

(10) $$f_{o}=Z_{mo}\times \left(r_{os}+r_{oi}\right)$$

Using equation (7), *f*_*o*_ becomes

(11) $$f_{o}=Z_{s}\times r_{so}+Z_{mo}\times r_{oi}$$

where the last substitution is necessary for maintaining detailed balance.

The mean lifetime (τ) of state is given by the ratio of the unnormalized occupancy of the state to the probability flux out of the state. Thus, the mean lifetime of state “s” and “o” is given as

(12) $$\tau_{s}=\frac{Z_{s}}{f_{s}}=\frac{1}{r_{sc}+r_{so}}$$

(13) $$\tau_{o}=\frac{Z_{mo}}{{{{Z_{s}\times r}_{so}+Z}_{mo}\times r}_{oi}}$$

Next, we write the following equations for modeling r_sc_, r_so_, and r_oi_ as functions of p and v (note that, like the equations for occupancies, one can use different functional forms).

(14) $$r_{sc}=\frac{r_{1}}{\exp^{{\left(-\frac{p-r_{2}}{r_{3}}\right)}^{2}}}-r_{4}$$

(15) $$r_{so}=r_{5}\times \exp^{\left(-r_{6}\times v\right)}$$

(16) $$r_{oi}=\left(r_{7}\times \exp^{\left(r_{8}\times p+r_{9}\right)}\right)\times \exp^{\left(r_{10}\times v\right)}$$

Where r_x_ are the unknown parameters that can be determined by substituting Equations (14), (15), (16) into Equations 12 and 13 and fitting the resulting equations to the mean lifetimes of state “s” and “o” as functions of pressure and voltage. The resulting model fits the experimental values and are displayed in Figure 6, *E* and *F*, and the parameter values are listed in Table 3. Rates r_cs_, r_os_, and r_io_ are obtained from the relationship between unnormalized occupancies and ratio of transition rates (Equations (6), (7), (8a)a). Rates r_ci_ and r_ic_ are constrained by the unnormalized occupancy of state “i” (Equation 8b). One can use an arbitrary value or function for one of these rates and get the other from Equation 8b. For simplicity, we take r_ci_ = 0.1 ms^−1^. Thus,

(17) $$r_{ic}=\frac{r_{ci}}{Z_{I}}$$

**Table 3:** Parameter values for Piezo1 rate constants between different states

Parameter	Values	Units
r_1_	0.6772619	ms^−1^
r_2_	25.283892	mm Hg
r_3_	350.94011	mm Hg
r_4_	0.6029228	ms^−1^
r_5_	0.2022486	ms^−1^
r_6_	0.0756368	mV^−1^
r_7_	7.4682 × 10^−9^	ms^−1^
r_8_	0.2580212	mm Hg^−1^
r_9_	5.3280 × 10^−3^	ms^−1^
r_10_	1.0472 × 10^−2^	mV^−1^

Note that we cannot use the mean shut time to obtain r_ci_ or r_ic_ explicitly as it is not possible to separate the dwell times of state “c” and “i” because the mean current levels are 0 pA in both cases.

The ratios of transition rates between neighboring states as functions of pressure are shown in Figure 6*A*. The increase in the ratio r_cs_/r_sc_ (the probability flux from “c” to “s” with respect to the probability flux from “s” to “c”) is consistent with the increase in the occupancy of state “s” with increasing the pressure. This, together with the fact that r_ic_/r_ci_ decreases, implies that the occupancy of state “c” decreases significantly with applied negative pressure (consistent with the pressure-dependent openings of the channels). The increase in the occupancy of “i” at the expense of “s” is somewhat compensated for the decrease in r_oi_/r_io_ as pressure is increased. The decrease in r_oi_/r_io_ and increase in r_so_/r_os_ also indicate that the channel spends more time in state “o” with more applied pressure. The slight dip in the occupancy of state “o” around −30 mmHg given by the model (Fig. 6*C*) is also evident from the slight decrease and increase in r_oi_/r_io_ and r_oi_/r_io_, respectively.

**Figure 7:**
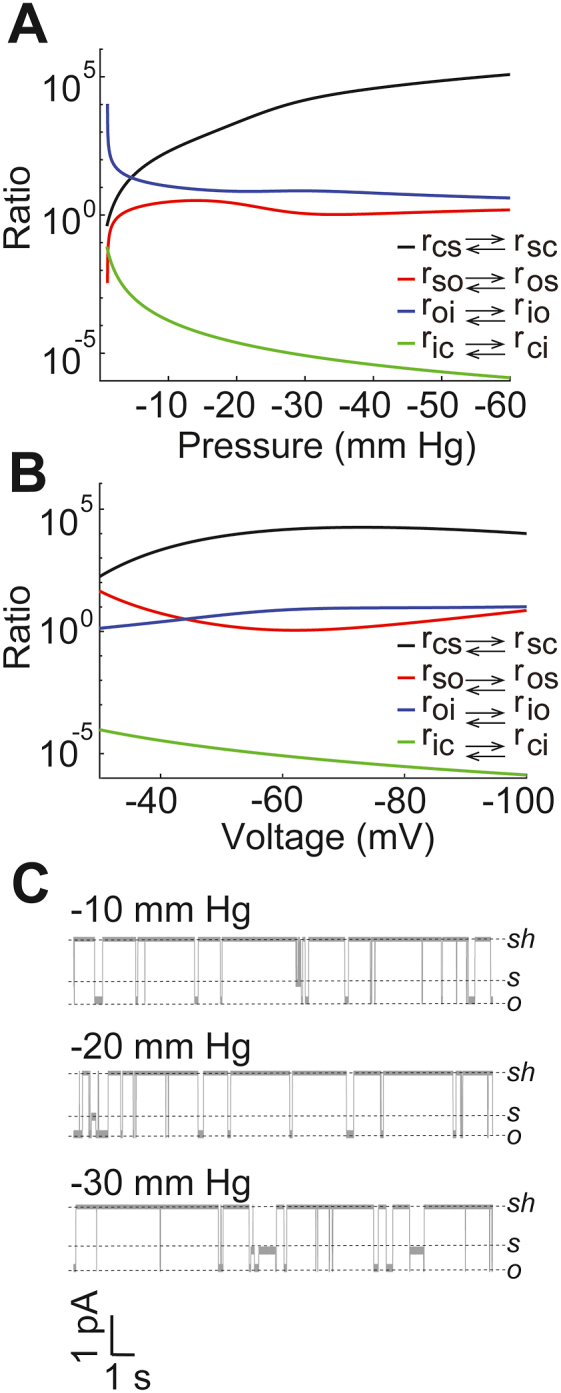
**Simulation of Piezo1 transition rates and kinetics as a function of pressure and voltage.** The ratio of transition rates between two neighboring states as functions of (*A*) pressure and (*B*) voltage. *C*, simulated time-traces at −60 mV membrane potential at −10 mm Hg (*top*), −20 mm Hg (*middle*), and −30 mm Hg (*bottom*) pressure.

The ratios of transition rates between neighboring states as functions of pressure (Fig. 7*A*) differ from states as functions of voltage (Fig. 7*B*) in that r_so_/r_os_ decreases and r_oi_/r_io_ increases at more negative membrane potential that decreases the time the channel spends in state “o.” The dip in r_so_/r_os_ around −60 mV indicates that the channel spends more time in state “s” at this potential. The increase in r_oi_/r_io_, together with the relatively small increase (with respect to the change as a function of pressure) in r_cs_/r_sc_, translates into higher total occupancy of state “c” and “i” at more negative potentials.

In Figure 7*C*, we show sample time traces for a single-channel constructed by the model at different pressure values. The channel was simulated stochastically as described in the methods section with no current (0 pA) when gating in state “c” and “i,” −1.11 pA current when gating in state “s,” and −1.54 pA when in state “o.” These values are selected based on the average values from experimental data (Figs. 1 and 2). We also add random noise drawn from uniform distribution over the interval [-0.091–0.091], chosen according to the root mean squared error in the current through the channel when gating in state “o.” In the future, such stochastic modeling can be used to study the effect of a single or multiple Piezo1 channels on the function of a whole cell.

## Discussion

In this study, we show that the direct application of negative pressure (and positive pressures) evokes single-channel activity of Piezo1 with distinguishable SCLs and open state. We noticed that the transitions to sublevels could be achieved either from the shut-state (sh → s) or open-state (o → s). However, sub-levels were more frequent and experimentally observed when visited from an open-state. Furthermore, these SCLs are an inherent permeation property of Piezo1 channels as shown by two distinct methods, (i) heterologous expression of Piezo1 in cells, (ii) protein purification and subsequent reconstitution in lipid bilayers.

For Piezo1, the identification and analysis of SCLs were not highlighted before. Other ion channels, such as voltage-gated potassium channels, are well studied in terms of their subconductance states. The homo-tetrameric potassium channels are assembled with four identical subunits, and so the classical interpretation has been that the conduction pathway is not formed unless all four subunits are in their active positions, leading to a shut- (all four subunits resting) or open-state (all four subunits active). This hypothesis agreed with the molecular interpretation of the open probability of n^4^, originally introduced by Hodgkin and Huxley in 1952 (38). The prevalent hypothesis is that four *n* particles (or four pore-forming subunits) are needed to be in the correct position to open the potassium channel pore and subsequent ion conduction. The simple two conductance level of potassium channels was experimentally challenged by Chapman *et al.* (21), when they described intermediate conductance levels in delayed rectifier potassium channel (drk1). In addition to short lived, longer-lived subconductance levels in drk1 were also observed that exceeded the filter risetime, giving confidence that those intermediate current levels were indeed the result of a channel conformation with a smaller conductance.

We apply a similar approach here to study gating kinetics and transitions of Piezo1 from different states. Piezo1 is a homo-trimeric ion channel with a central ion-conducting pore. Unlike potassium selective ion channels, where the selectivity filter sequence is well established (amino acids TVGYGD) (39), nonselective cation channels like Piezo1 lack the consensus selectivity filter sequence. While all three subunits are needed to form a central ion-conducting pore in Piezo1((2, 11, 40, 41)), there is no structural evidence of various Piezo1 pore conformations such as open pore *versus* collapsed or inactivated pore, which is overwhelmingly available for potassium channels such as KcsA (42, 43, 44). While there is no structural evidence to account for subconductance states in Piezo1 channels, it is highly likely that these channels follow a “unipore hypothesis” where sub-states, along with closed and main open state of channels, result from different conformational states of a single pore, and not the multipore (26). It is conceivable that one or two subunits might experience collapse or change of conformation at constant higher pressures, thus allowing only fraction of ionic current to pass, while the fully conductive state is achieved when all three subunits are in non-collapsed or active conformation. Our functional data suggest for the first time that sustained negative pressure (suction stretch) destabilized the fully open-state and allowed Piezo1 to dwell into subconductance state with ∼30 to 40% reduced single-channel currents than the fully open-state.

### Four-state kinetic scheme for Piezo1

While single-channel models for Piezo1 have been developed before (27, 28, 45), the transition rate for those models are given at discrete pressure values. That is, data obtained at fixed pressure was used to obtain the rates. This prevents using the model to investigate how one or more Piezo1 channels affect the function of a cell as the cell experiences a continuous change in pressure. Our model overcomes this shortcoming by formulating the transition rates as continuous functions of pressure. Our data also show that not only the amplitude of current through the channel changes due to change in the driving force when the membrane potential changes, but the gating properties of the channel are also impacted. This voltage-dependence of the channel’s kinetics was not considered in the previous models. Furthermore, this is the first model that incorporates the sub levels of Piezo1 channel, which could be of significance to the cell’s function where hundreds of molecules are expressed.

Our data and conclusions closely mirror independent studies published by Wijerathne *et al.* (27), that is, Piezo1 shut state (and dwell times) is highly dependent on applied pressure; however, the previous studies did not report the occurrence of subconductance state. This could be due to the key differences in data acquisition, analysis, and presentation between our studies and Wijerathne *et al.* First, the data acquired by Wijerathne *et al.* is at a constant voltage of V = −90 mV. We showed here that the occupancy of open and sub state of Piezo1 decreases sharply from −40 to −100 mV (Table 1 and Fig. 5). Based on our observations, it is evident that at −90 mV, the occurrence of sub-states was less frequent and therefore they were unable to detect subconductance states in their histograms. Second, they did not construct histograms on log scale (Count on y-axis) that could help in identifying sublevels at −90 mV, like we observed at −100 mV when histograms’s y-axis were constructed at log scale (Fig. 5). Close visual inspection of their Figure 1*A* (27) shows distinct sublevels which then appear to be increasing in frequency from 0 mm Hg to −90 mm Hg pressure. The visual observation of their Figure 1*A* is in line with our data and analysis performed in Figures 2 and 4. Third, the focus of their studies was not the identification of sub-states, and they did not present data at voltages where we observe the most occupancy of open- and sub-states. Fourth, the permeant ion in their case is only K^+^ (they used 140 mM KCl in pipette solution), while in our studies, the permeant ions are Na^+^, Ca^++^, and Mg^++^ (130 mM KCl, 1 mM CaCl_2_, and 1 mM MgCl_2_ in pipette solution). It is possible that the permeant ions (either monovalent or divalent) can modulate the occupancy of sublevels by either stabilizing or destabilizing the fully open or shut state. Further in-depth studies are required to address this interesting observation. Lastly, the acquisition and filtering frequency used in their studies are different than ours. While their acquisition frequency is higher (500 kHz) than ours (20 kHz), the filtering frequency is 10X in both the cases (they filter at 50 kHz and we filter at 2 kHz). While their data points are filtered every 20 μs and ours at 50 μs, it is unlikely that Piezo1 goes into these ultra-fast open- and sub-state transitions where we miss acquiring data points that are critical for analysis (for that to happen, the mean open- and sub-state times should be less than 50 μs and we observe dwell times >1 ms, Fig. 4). Another evidence that we observe the true subconductance states (possibly due to a change in channel pore conformation) and not the artifact of filtering is noted with the increase in sublevel occupancy with applied pressure and not with applied voltages. If we were to miss the transitions, we would not have seen an effect of pressure at similarly filtered data within and in between various recordings.

Another advantage of our model is that it lends itself to extension conveniently, which allows incorporating future observations. For example, if the channel gating is found to be dependent on another variables (such as permeant ion or small molecule), the occupancies and rates can be tweaked to incorporate the dependence on the new variable without affecting the fits to the kinetics as functions of pressure and voltage dependence. Similarly, if stronger evidence supporting additional sub levels emerge, one can simply split the total P_o_ into more than two occupancies (as done here) to incorporate new sub levels and derive the transition rates based on the mean lifetime data of the newly detected sub levels. To summarize, our model not only allows for future extension but is also well-suited for investigating how Piezo1 affects cell’s function at the sub-cellular and whole-cell levels.

Here, we put forth the hypothesis (based on experimental data and modeling) that the occurrence of Piezo1 sub-states at steady-state physiological conditions could serve as ionic influx regulatory mechanism (calcium and/or sodium) to avoid unwanted cell depolarization or downstream signaling events when cells experience constant forces. This regulation of ionic flux *via* Piezo1 may play a role in slower physiological process such as bone formation and homeostasis and cell division and fate determination (3, 46, 47, 48, 49, 50), where steady-state Piezo1 channel population becomes mildly dormant by dwelling into sub-levels. We also remark that so far, the mechanotransduction of Piezos (both Piezo1 and Piezo2) are physiologically studied for acute and fast process such as sense of touch and tactile pain (51, 52), proprioception (53, 54), red blood cell volume regulation (4, 55), hence there are not many examples and mouse models of Piezo1 linking ion permeation at steady-state to physiologically relevant processes. Though the experiments presented here were acquired under constant pressure, it is conceivable that our hypothesis is extended to other mechanical stimuli at steady-state, such as shear stress and prolonged tension in the membrane. Our single-channel kinetics data in future can be used to develop cellular models where the activity of hundreds of Piezo1 molecules could be predicted under various pressures, sheer stress, and voltage conditions, as well as in the presence of pharmacological agents and Piezo1 modulators.

## Experimental procedures

### Cell culture and transient transfection

HEK293T^ΔP1^ cells (PIEZO1 KO cell line) were purchased from ATCC. Cell lines were confirmed to be mycoplasma-negative when cultures were started in the lab. HEK293T^ΔP1^ cells were maintained in Dulbecco’s modified Eagle’s medium supplemented with 10% fetal bovine serum, penicillin, and streptomycin. Transient transfection was performed using Lipofectamine 3000 (Thermo Fisher Scientific) according to the manufacturer’s instructions.

### Electrophysiology

Transfected HEK293T^ΔP1^ cells were visualized using Nikon eclipse Ti2 microscope and C11440 Orca-Flash 4.0 LT digital camera (Hamamatsu). Cell-attached recordings of pressure-activated currents in HEK293T^ΔP1^ cells expressing the WT Piezo1 were performed using experimental setup based on an Axopatch 200B amplifier and Digidata 1550B digitizer (Molecular Devices). Currents were acquired with pClamp 10.7 software (https://www.moleculardevices.com/products/axon-patch-clamp-system) and were recorded at a sampling frequency of 10 or 20 kHz. Recording patch pipettes of borosilicate glass were pulled and fire-polished to a tip resistance of 4 to 6 MΩ. The bath solution contained (in mM) the following: 140 KCl, 10 Hepes, 1 MgCl2, 10 glucose, pH 7.3 (pH adjusted with KOH). The pipette solution contained (in mM) the following: 130 NaCl, 5 KCl, 10 Hepes, 10 TEA-Cl, 1 CaCl2, 1 MgCl2, pH 7.3 (pH adjusted with NaOH). The bath electrode was grounded while the pipette electrode was active such that at positive applied potential, cations moved from pipette to bath (inward currents). Mechanical stimulation was applied *via* recording pipette using a high-speed pressure clamp system (ALA Scientific Instruments). Single-channel events are shown as downward deflection representing inward currents acquired at −60 mV (to match conventional direction of flow). The single-channel data was analyzed using Clampfit 10.7.

### Analysis and statistics

All-point current-amplitude histograms of the single-channel data (ranging from 20 to 30 s stretch) were constructed in Clampfit or custom algorithms written in Matlab. No thresholds were set to identify sublevels or fully open state and the analysis was done in an un-biased manner. All-point current-amplitude histograms (either linear or log scale) were constructed to identify peaks between open and shut states. Further, Gaussian functions with 2, 3, and 4 parameters were used to fit the data. The best fits were obtained with 4 parameters, attributing to shut-, open-, and two major sub-states of the channel. The single-channel current values were extracted after fitting the Gaussian function to the data. Each mean value presented is an average of at least 12 or at most 24 individual patch recordings. Eight to twelve individual coverslips with transfected cells were used to acquire single-channel Piezo1 data. Open probability of the channel was calculated exclusively from the records, where at least 20 s of data was recorded both before and after pressure application. The number of channels in the patch is determined by fitting a Gaussian curve to all-point current-amplitude histograms to individual open peak. At least a 20 to 30 s stretch of recordings were analyzed to observe for consecutive channel openings. Group data (mean lifetime and occupancy bar graphs) are presented as mean ± SD where ∗*p* < 0.05; ∗∗*p* < 0.01, ∗∗∗*p* < 0.001, ∗∗∗∗*p* < 0.0001, not significant (ns) *p* > 0.05. Unpaired two-tailed t-tests were used for comparison between the two groups in question. One way ANOVA was performed for more than two groups in question.

### Numerical methods for modeling

Modeling the gating kinetics of an ion channel requires idealized traces representing the conductance level in which the channel is gating as a function of time. To idealize the raw time traces, we used an in-house software called TraceSpecks (56), which uses maximum likelihood formalism for separating signal from a noisy and drifting background. A key advantage of this formalism is that it does not require an *a priori* assumption about the number of conducting states in a time trace. The number of conducting states results automatically after maximizing the likelihood score. After removing experimental biases and noise from the raw time trace, an idealized trace revealing various conducting states is generated. TraceSpecks also compute dwell times, mean lifetimes, and occupancies of different conducting states and open probability (P_o_) of the channel, which can be later used to develop single-channel model. The results from TraceSpecks were verified by processing traces using QUB (57).

After the processing and idealization step, all-point current-amplitude histograms, dwell-time histograms, and other statistical analysis was performed in Matlab. The current-amplitude histograms, dwell-time histograms, P_o_, state occupancies, and mean lifetimes at different pressure and voltage values were fitted by analytical functions or a Markov chain model using the least square fitting method. Matlab’s function “fmincon” was used to minimize the residual sum of squares.

To generate time traces representing the gating of a single Piezo1 channel from the model, we employed the procedure outlined in previous publications (33, 58). Briefly, if the channel is in state x at time t, the probability with which it makes transition to a neighboring state y or remains in state x, within the sufficiently small time interval dt is given by P_xy_ = r_xy_ dt where r_xy_ is the transition rate from state x to y and $P_{\text{xx}}=1-\;\sum_{i}r_{\text{xi}}\text{dt}$, where the sum is over all states in the model. r_xi_ = 0 for the states not directly connected to x. The unit interval is divided into j subintervals of length P_xj_, where j represents the states to which the channel can make transition (including the current state x). If a random number drawn from a uniform distribution over the interval dt falls into the subinterval P_xi_, the corresponding transition is performed. For example, if the channel is in state “c” (Fig. 5*A*), the three possible direct transitions are to “s,” “i,” or staying in state “s.” The unit interval is divided into three subintervals [{0, P_cs_} {P_cs_, P_cs_+ P_ci_} {P_cs_+ P_ci_, 1}]. If the random number falls between the first subinterval, the channel makes transition to state “s.” This approach is similar to the Gillespie algorithm. However, the time interval is kept fixed and small enough for the linear dependence of P_xx_ on the time interval to remain valid.

## Data availability

Piezo1 clone, reagents, experimental and modeling protocols, and the data analysis are contained within the article and further available upon request.

## Conflict of interest

The authors declare that they have no conflicts of interest with the contents of this article.
